# Synthesis and application of a multifunctional poly (vinyl pyrrolidone)-based superabsorbent hydrogel for controlled fertilizer release and enhanced water retention in drought-stressed *Pisum sativum* plants

**DOI:** 10.1038/s41598-024-76255-7

**Published:** 2024-11-12

**Authors:** Mohamed Mohamady Ghobashy, Mohamed A. Amin, Abeer E. Mustafa, Mahmoud A. El-diehy, Basem Kh. El‑Damhougy, Norhan Nady

**Affiliations:** 1 Radiation Research of Polymer Department, National Center for Radiation Research and Technology (NCRRT), Atomic Energy Authority, P.O. Box 29, Nasr City, Cairo Egypt; 2 Department of Botany and Microbiology, Faculty of Science, Al-Azhar University, Cairo, 11884 Egypt; 3 Department of Botany and Microbiology, Faculty of Science (Girls), Al-Azhar University, Nasr City, 11884 Cairo Egypt; 4 Department of Chemistry, Faculty of Science, Al-Azhar University, Cairo, 11884 Egypt; 5 Polymeric Materials Research Department, Advanced Technology and New Materials Research Institute (ATNMRI), City of Scientific Research and Technological Applications (SRTA-city), New Borg El-Arab City, 21934 Alexandria Egypt

**Keywords:** Multifunctional superabsorbent, Fertilizers carrier, Water retention, *Pisum sativum*, Sustainable agriculture, Plant sciences, Chemistry

## Abstract

Water scarcity poses a significant challenge to agricultural production, prompting the development of sustainable technologies to optimize water resource utilization. This study focuses on the synthesis and application of a multifunctional poly (vinylpyrrolidone); PVP-based superabsorbent hydrogel (SAH) for controlled release of essential fertilizers (nitrogen, phosphorus, and potassium) and enhanced water retention in soil. The hydrogel was prepared via a facile one-step approach and compared to a control soil without hydrogel amendment. The reaction was initiated in the presence of poly(vinylpyrrolidone) (PVP) to produce a PVP-based copolymer hydrogel. The hydrogel was then subjected to a basic treatment using either sodium hydroxide (hydrogel #1) or potassium hydroxide (hydrogel #2). The PVP-based SAH exhibited excellent swelling capacity, water retention, and fertilizer release properties. When applied to *Pisum sativum* plants under drought stress, the hydrogel significantly improved soil moisture levels, nutrient availability, and plant growth parameters compared to the control. The hydrogel #2-amended plants demonstrated enhanced biomass, chlorophyll content, and photosynthetic efficiency, highlighting the hydrogel’s effectiveness in mitigating the adverse effects of drought stress. These findings demonstrate the potential of the PVP-based SAH as a promising strategy for sustainable agriculture, offering using readily available and inexpensive raw materials, suggesting a relatively low-cost and scalable production process. Furthermore, the hydrogel facilitates water conservation, controlled nutrient delivery, and improved plant performance under drought stress conditions.

**Supplementary Information:**

The online version contains supplementary material available at 10.1038/s41598-024-76255-7.

## Introduction

Water plays a critical role in agricultural production. The agricultural sector consumes about 70% of the available water resources. Water shortage is an important global issue that is aggravated due to the increased pressure on water resources in response to climate change and the continuous increase of population, it affects agriculture and causes soil desertification and salinization^1^. Moreover, water shortage obstacles to the sustainable development of agriculture as well as food security^2^. In such a context several agricultural systems have been developed to promote technologies that could optimize the exploitation of water resources including precise irrigation^3^, applying modern irrigation methods^4,5^, mulching^6^, deficit irrigation^7,8^, and water harvesting^9^, etc. One of the researchers’ targets is to find a method/material that can keep water and nutrition for a long time in the soil to help agriculture under water stress.

Superabsorbent hydrogels (SAHs) are a practical approach to regulating water consumption in agriculture by retaining moisture in the soil and reducing irrigation water consumption^2,10^. The SAHs are three-dimensional cross-linked hydrophilic polymers that can absorb water hundreds to thousands of times their masses^11^. They have a high swelling capacity and high swelling rate^10,11^. When water comes into contact with one of these polymeric chains of SAHs, it is drawn into the molecule by osmosis. Water rapidly migrates into the interior of the SAHs network where it is stored. As the soil dries out, 95% of the absorbed water is released from the polymer back into the soil^11^. Moreover, SAHs can improve the physical properties of soil and lower the death rate of plants. Furthermore, loading fertilizers to SAHs can enhance fertilizer retention in soil, control their release, and hence avoid unnecessary environmental pollution caused by conventional fertilizers^12^. In general, SAHs play a crucial role in soil structure and fertility. They act as binding agents, cementing soil particles together into stable aggregates. This improves soil aeration, water infiltration, and root penetration. SAHs also contribute to cation exchange capacity, buffering soil pH, and retaining nutrients and water in the soil profile^13,14^.

The superabsorbent hydrogel developed in this study is based on the copolymerization of polyvinylpyrrolidone (PVP) and acrylic acid (AA) monomers. PVP is a well-known synthetic polymer with excellent biocompatibility, hydrophilicity, and water-soluble properties. The incorporation of AA introduces carboxyl groups (-COOH) into the hydrogel structure, which can enhance the swelling capacity and water retention abilities of the material. To further improve the hydrophilic nature and swelling behavior of the PVP-based hydrogel, we have employed an alkali treatment step. By partially neutralizing the carboxyl groups in the hydrogel, the introduction of ionic carboxylate (–COO⁻) groups can significantly increase the hydrophilicity and water absorption capacity of the material.

Generally, SAHs are classified into three types based on the sources of raw materials, namely natural (polysaccharide derivatives), semi-artificial (cellulosic primitive derivatives), and artificial polymers. Natural SAHs are attractive due to their renewability and biodegradability^15^. They can be degraded by natural biological processes, including the actions of enzymes, micro-organisms, and water, transforming into harmless and simple compounds that are environmentally safe. Amongst them, polysaccharides and proteins have been employed to prepare SAHs due to their availability on a large scale as feedstock^16^, renewability, and biodegradability. Polysaccharides are naturally polymeric carbohydrate molecules composed of monosaccharide units joined by glycosidic bonds. The structure varies from linear to highly branched. Examples of polysaccharides include cellulose and chitin^1^. The performance of SAHs can be enhanced by various methods such as increasing their hydrophilicity^2^ and constructing specific network structures, such as interpenetrating polymer network (IPN)^3^, semi-interpenetrating polymer network (semi-IPN)^4^ and copolymer network^5^. The capability of the hydrogels to absorb water is because of the hydrophilic functionalities attached to the polymeric backbone, cross-linker is added to the gel to prevent dissolution of the gel^6^. If the superabsorbent polymer has both ionic hydrophilic groups (carboxyl, sulfonic acid group, tertiary amine group, etc.) and non-ionic hydrophilic groups (hydroxyl, amide group, ester group, etc.), its salt tolerance, water absorption rate, and gel strength will be significantly improved^7^. If the superabsorbent polymers have a multi-porous structure, it can increase the contact area with water to improve the water absorption rate^8,17^.

Cellulose is the most abundant natural organic substance in the world. It comes from a wide range of sources, including cotton, wood, and plant straw. Cellulose is a kind of water-absorbent material containing multiple hydroxyl groups and has a certain water absorbency capacity. In addition, it has a large specific surface area, biocompatible, biodegradable, non-toxicity, low cost, and renewable^7,9^. Nevertheless, the absorbency of neat cellulose is very low (below 30 g/g in distilled water) owing to its highly dense crystalline structure^10^. Water absorbency can be enhanced by destroying the dense crystalline structure via modification of cellulose^11,17^. Various cellulose derivatives, such as cellulose acetate, carboxymethylcellulose, hydroxyethylcellulose, and so on, have been utilized as polymer backbones in SAHs expected to achieve biodegradability^12^.

Hydroxyethylcellulose (HEC) is a modified cellulose synthesized by reacting ethylene oxide and cellulose with an alkaline catalyst under carefully controlled conditions^16^. Since the HEC chains have abundant reactive hydroxyl groups, they can be modified by graft polymerization with hydrophilic vinyl monomers to produce materials with desirable properties^17^. While Carboxymethyl cellulose (CMC) does exhibit extensive hydrogen bonding as mentioned in previous work^18^, this can also lead to increased viscosity and reduced swelling ability compared to HEC. HEC, on the other hand, has a lower degree of hydrogen bonding, which can result in better swellability and water absorption capacity. HEC is a nonionic polymer, whereas CMC is an anionic polymer. The lack of charge on HEC can be advantageous in certain applications where the interactions between the hydrogel and other charged species (e.g., ions, proteins) need to be minimized. Also, HEC generally has better thermal stability compared to CMC, which can be important for applications involving elevated temperatures or thermal processing. Moreover, the nonionic nature of HEC can make it more compatible with a wider range of other polymers, additives, and active ingredients in the hydrogel formulation.

Many studies have reported on HEC-based superabsorbent composites. Hydroxyethyl cellulose g-poly (acrylic acid)/attapulgite (HEC-g-PAA/APT) superabsorbent composites were synthesized through the graft polymerization of HEC, partially neutralized acrylic acid (AA), and attapulgite (APT) in aqueous solution^17^. Water absorbency and water absorption rate of the prepared superabsorbent composites were enhanced by the introduction of 5 wt% APT into HEC-g-PAA polymeric network^18^. Also, superabsorbent polymers were prepared entirely by graft copolymerization of polyacrylamide (PAM) onto HEC using potassium persulfate (KPS) as an initiator, and N, N′-methylenebisacrylamide (MBA) as a crosslinker, in an aqueous solution that showed good swelling capacity^19^. Recently, a hydrogel of HEC-g-P (AA-co-AMPS)/laterite was fabricated by aqueous solution polymerization. The SAHs had good water retention and re-swelling properties at different temperatures, and fast water absorption rate, and reached swelling equilibrium at 5 min^7^. Durpekova et al. prepared a hydrogel of HEC and sodium carboxymethylcellulose (CMCNa) mixtures in the presence of citric acid (CA) as crosslinker. The prepared hydrogel showed good swelling properties as well as a source of a nutritive whey agent for plants and a means of absorbing and releasing fertilizers in soil^19–21^.

Arabic Gum is a natural polysaccharide that can act as a thickening, emulsifying, and stabilizing agent in hydrogel formulations due to its non-toxic and biocompatible nature^22^. It dissolves easily in water, yielding transparent solutions that range in color from very pale yellow to orange and have a pH of ~4.5. The grafting of acrylic monomers (e.g., acrylic acid and acrylamide) onto the GA backbone seems to be the preferred strategy by many authors since it allows obtain mechanically stable hydrogels, often endowed with superabsorbent properties^23,24^.

Another important required property of SAHs is to be slow-release or controlled-release for fertilizers that can release nutrients gradually over an extended period, rather than conventional providing immediate, full-dose fertilizers. This provides advantages over conventional fertilizers including improved nutrient efficiency in which slow-release fertilizers provide a steady supply of nutrients to plants over time, reducing the risk of nutrient deficiencies or excesses as well as this leads to better uptake and utilization of nutrients by plants, reducing the potential for nutrient leaching or runoff. Also, the gradual nutrient release from slow-release fertilizers means fewer nutrients are lost to the environment through leaching, volatilization, or runoff. This helps to reduce the risk of water pollution, soil degradation, and greenhouse gas emissions associated with conventional fertilizers^25^. Moreover, decreased maintenance requirements in which slow-release fertilizers typically need to be applied less frequently than conventional fertilizers, reducing the time and labor required for fertilizer application. Improved plant health and growth in which the steady supply of nutrients from slow-release fertilizers can promote healthier root development, better overall plant vigor, and potentially higher yields or improved quality of crops. This can be especially beneficial for plants that require a consistent, balanced nutrient supply for optimal growth and performance. Moreover, reduced risk of fertilizer burn in which conventional fertilizers can sometimes cause fertilizer burn, which is the damage or death of plants due to the application of too much fertilizer at once^25,26^.

In this work, a multifunctional poly(vinylpyrrolidone) (PVP)-based superabsorbent hydrogel (SAH) for controlled release of essential fertilizers (nitrogen, phosphorus, and potassium) and enhanced water retention in soil was synthesized. The present hydrogel formulation incorporates several key components, each serving a specific purpose: Polyvinylpyrrolidone (PVP) forms the primary polymer network, providing the base structure for the hydrogel. Arabic gum acts as an emulsifier and stabilizer, enhancing the overall stability of the hydrogel structure. Hydroxyethylcellulose (HEC) and polyethylene oxide (PEO) serve as thickening agent and rheology modifier, contributing to the hydrogel’s water retention capabilities. Acrylic acid (AAc) introduces additional hydrophilic groups, improving water absorption, while N, N′-methylenebisacrylamide (MBA) acts as a crosslinking agent, enhancing the structural integrity of the hydrogel. The hydrogel was subsequently treated with NaOH or KOH to enhance its swelling and water retention properties. The treated hydrogels were characterized and applied in agricultural of *Pisum sativum* under water stress. The swelling degree, water retention, gel fraction, and scanning electron microscope (SEM) imaging were used for determine the effect of the used treatments on the hydrogels. Also, the fertilizer release behavior was determined and discussed.

## Experimental

### Materials

Polyvinylpyrrolidone (PVP, purity > 99%) K90, methylenebisacrylamide (MBA, purity > 98%), and persulfate initiators (purity > 97%) were purchased from Sigma-Aldrich. Arabic Gum (purity > 98%) was obtained from Kerry Group. Hydroxyethylcellulose (HEC, purity > 97%) was obtained from Ashland, and polyethylene oxide (PEO, purity > 98%) was obtained from Dow Chemical Company. Acrylic acid (AAc) was purchased from BASF. Distilled water was used in the preparation process.

### Preparation of a multifunctional PVP-based superabsorbent hydrogel

A 5% w/v polyvinylpyrrolidone (PVP) solution was prepared using distilled water as the solvent. To this solution, 1% w/v of Arabic gum was carefully added dropwise with continuous stirring to ensure thorough mixing. Next, a solution containing 1% w/v of both hydroxyethylcellulose (HEC) and polyethylene oxide (PEO) was prepared and subsequently added dropwise to the PVP/Arabic gum mixture, again with constant stirring to maintain homogeneity. Then, a solution of acrylic acid (AA) was prepared by dissolving and neutralizing it with 0.1 M potassium hydroxide (KOH), along with 200 mg of N, N′-methylenebisacrylamide (MBA). This AA solution was introduced dropwise into the PVP/Arabic gum/HEC/PEO mixture with continuous stirring to ensure a uniform reaction. Following the addition of the AA/MBA solution, an aqueous mixture containing persulfate initiators was also added dropwise to the reaction mixture. This mixture was then heated to 50 °C and maintained at this temperature for 30 min under a nitrogen atmosphere to eliminate any dissolved oxygen that could interfere with the reaction.

**Fig. 1:**
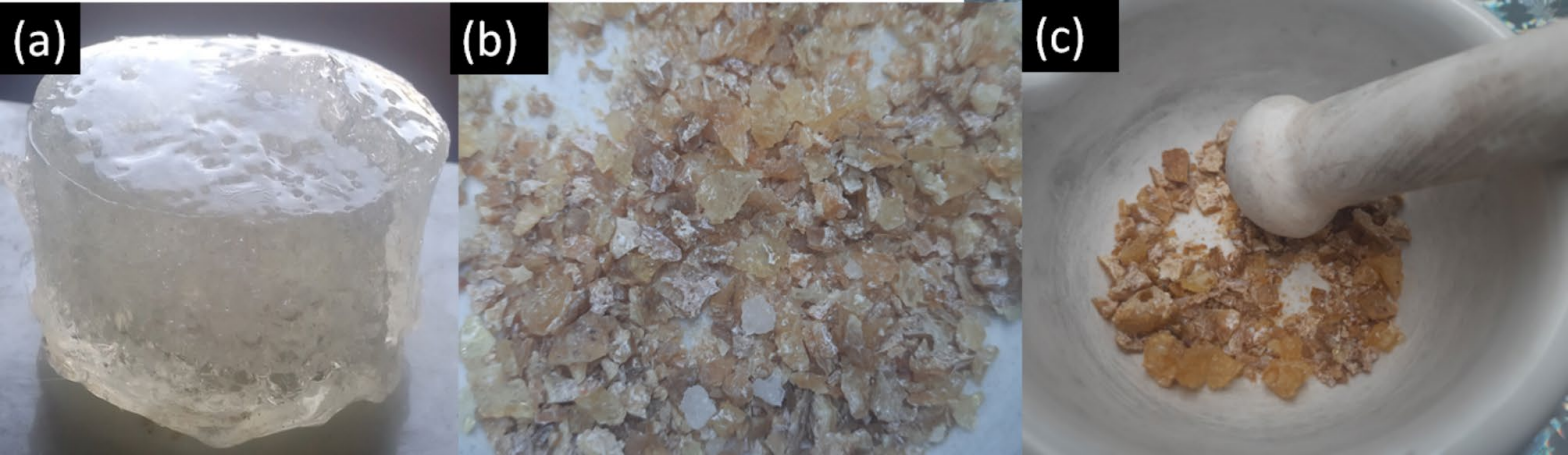
The swelled hydrogel as prepared (**a**), the dried and grinding hydrogel, (**b**) and (**c**), respectively.

The resulting product, a hydrogel referred to as PVP/AAc, was formed as shown in Fig. 1a. The hydrogel was then cut into small pieces, as depicted in Fig. 1b. These pieces were dried in an oven set at 50 °C, as shown in Fig. 1c.

To further process the hydrogel, it was treated in a three-neck flask. Either 20 ml of 1 M NaOH or 1 M KOH was added dropwise, with continuous stirring, to the hydrogel at a temperature of 70 °C. This step aimed to treat the hydrogel, resulting in two variants: one treated with NaOH (hydrogel#1) and the other with KOH (hydrogel#2). After a treatment period of 3 h, during which the hydrogel was stirred continuously to ensure complete reaction, the hydrogel samples were thoroughly washed to remove any residual chemicals and then dried again in an oven at 50 °C.

### Procedure of the three-fertilizer release (urea (N), K, and P)

To investigate the controlled release of three essential fertilizers—urea (N), phosphorus (P), and potassium (K)—within a hydrogel matrix, a systematic procedure was employed.

Initially, dry hydrogel samples were accurately weighed to ensure consistent loading and subsequent analysis. These hydrogels were then immersed in saturated solutions of urea, phosphorus, and potassium, allowing for maximal absorption of these nutrients into the hydrogel network. This soaking process was maintained for 24 h, during which the hydrogels became fully swollen, indicating that the fertilizers were effectively loaded into the hydrogel matrix.

Post-absorption, the nutrient-laden hydrogels were carefully removed from the soaking solutions and left to dry at room temperature. This drying process was crucial to stabilize the hydrogels and to prepare them for the release experiments.

To assess the release profiles of urea, phosphorus, and potassium from the dried hydrogels, a series of controlled release experiments were conducted. Each dried hydrogel sample was placed in a vessel containing 20 ml of deionized water at room temperature. This aqueous environment simulates typical conditions under which the hydrogels might release their nutrients in a practical application, such as in soil.

The release process was monitored over time, with aliquots being systematically withdrawn from the vessel at predetermined intervals. These aliquots were analyzed to determine the concentration of each fertilizer released from the hydrogel matrix into the surrounding water. The analytical technique employed for measuring the released nutrients was Inductively Coupled Plasma (ICP, Germany) Spectroscopy.

### Swelling ratio (g/g)

The hydrogels were prepared in cylindrical form with dimensions of approximately 1 cm in diameter and 0.5 cm in height. The swelling ratio was measured using the tea bag method. Dry hydrogel samples (0.1 g) were enclosed in a tea bag (nylon screen mesh, 300 mesh) and immersed in 200 mL of distilled water at room temperature. At predetermined time intervals, the tea bag was removed, surface water was gently blotted with filter paper, and the swollen hydrogel was weighed. The swelling ratio was calculated using the equation:

$${\text{Swelling ratio}}\;\left( {{\text{g}}/{\text{g}}} \right){\text{ }}={\text{ }}\left( {{\text{Ws }} - {\text{ Wd}}} \right)/{\text{Wd}}$$

where Ws is the weight of the swollen hydrogel and Wd is the initial weight of the dry hydrogel.

### Water retention measurement

The swollen gel of known weight was placed at room temperature. The gel was weighed at different time intervals and the weight loss of water was calculated as follows:

$${\text{Water retention }}\left( \% \right)={\text{ }}\left( {{{\text{W}}_{\text{t}}}/{{\text{W}}_{{\text{max}}}}} \right){\text{ }} \times {\text{ 1}}00$$

where W_max_ and W_t_ are the weights of retained water inside the hydrogel at initial (maximum swelling) and time t, respectively.

### A field experiment was conducted to show the influence of a superabsorbent hydrogel #2 on *Pisum sativum* under water stress

#### Plant material and experiment design

The seeds of *Pisum sativum* L. variety Alderman (garden pea) were sourced from Sakkha research center, Agriculture Ministry, Kafr El-sheikh, Egypt. The study was conducted in El Gharbia Governorate, Egypt, utilizing sandy loam soil. The soil’s physical and chemical properties were analyzed at Ain Shams University, Faculty of Agriculture in Cairo, with results detailed in Table 1s. For the experiment, each seed of *Pisum sativum* was treated with 5 g of Hydrogel 2#. Details of the treatment process can be found in Supplementary file (Table 2s and Fig. 1s). Treatments were categorized into two groups: one treated with superabsorbent hydrogel #2, denoted as “Hydrogel,” and the other untreated, labeled as the “Control.” These treatments underwent two stress levels: 0% and 100%, designated as follows: Control at 0% stress (C1), Hydrogel at 0% stress (H1), Control at 100% stress (C2), and hydrogel at 100% stress (H2). To ensure plant viability under varying stress levels, watering schedules were adjusted accordingly: every 10 days for 0% stress (normal irrigation according to Egyptian agriculture guidelines) and every 20 days for 100% stress.

#### Morphological growth and yield traits

Plant samples were harvested at two stages: 46 and 60 days after sowing, and assessed for morphological and biochemical characteristics. Morphological assessments included shoot and root lengths, fresh and dry weights of plant parts, and leaf count. After 100 days after sowing, seeds weights, 100 seed weight, number of seeds, number of pods were assessed.

#### Biochemical analyses

Biochemical analyses encompassed chlorophyll a and b, total chlorophyll, carotenoids of leaves, phenol, proline, protein, carbohydrate of dry shoot and yield, hydrogen peroxide, malondialdehyde (MDA), and levels of antioxidant enzymes of terminal shoots. All the experimental procedures of Pigment and Carotenoid Identification, total phenolic compounds, free proline levels in plant tissues, extraction and quantification of catalase, peroxidase, and polyphenol oxidase enzymes, extraction and quantification of soluble carbohydrates, and extraction and quantification of water-soluble proteins are available in the Supplementary file.

Experimental research, including the collection of plant material, complies with relevant institutional, national, and international guidelines and legislation. A permission to collect seeds of *Pisum sativum* was obtained.

### Statistical assessment

Statistical calculations were performed using computer programs Microsoft Excel version 365 and statistical Minitab program (Version 18). Quantitative data were analyzed by parameter distribution between different plant samples using analysis of variance for one-way ANOVA with Tukey’s post hoc test at the 0.05 level. Artwork and figures were created using Microsoft Excel version 365 software and GraphPad Prism software (version 8).

## Results and discussion

### The swelling ratio and water retention of the modified hydrogels

**Fig. 2:**
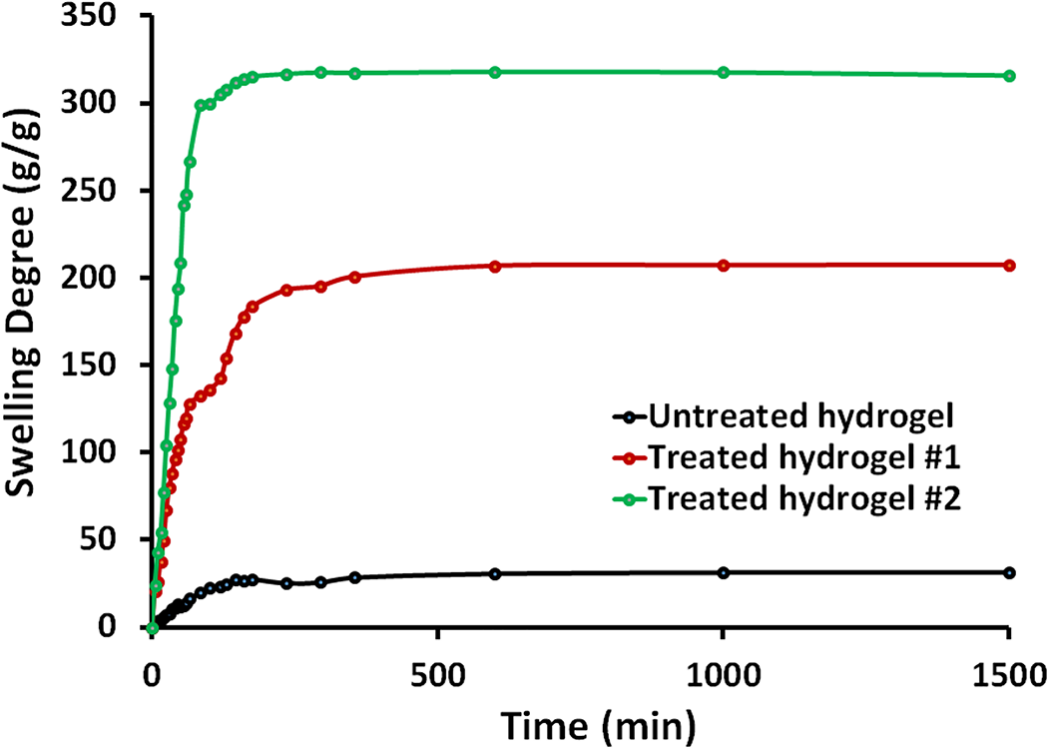
The swelling degree (g/g) as a function of time (min) of the prepared untreated hydrogel (black line), treated hydrogel #1 (red line), and treated hydrogel #2 (green line).

Figure 2 depicts the swelling degree (g/g) as a function of time (min) for the prepared untreated hydrogel, treated hydrogel #1, and treated hydrogel #2. The swelling degree is a measure of the amount of water absorbed by the hydrogel relative to its dry weight. The maximum swelling degree follows the order: treated hydrogel #2 (315 g/g) > treated hydrogel #1 (207 g/g) > untreated hydrogel (31 g/g). This significant increase in swelling capacity for the treated hydrogels can be attributed to the modification with NaOH (hydrogel #1) and KOH (hydrogel #2), which likely increases the hydrophilicity and ionic character of the hydrogel network, allowing more water to be absorbed. Figure 2 shows that treated hydrogel #2 reaches its maximum swelling of around 315 g/g within 175 min, while treated hydrogel #1 takes around 1500 min to reach 207 g/g swelling. The untreated hydrogel exhibits a relatively rapid swelling, reaching its maximum of 31 g/g within 1000 min, but this maximum value is significantly lower than the treated hydrogels. The alkaline treatment in hydrogel #2 introduced ionizable carboxylate groups (-COO-) along the polymer backbone. These negatively charged groups lead to increased electrostatic repulsion between the polymer chains, which in turn enhances the hydrogel’s ability to absorb and retain water molecules. The observed difference in swelling behavior between the two hydrogels can be attributed to several key factors. Hydrogel #2, treated with KOH, contains larger K + ions compared to the Na + ions from the NaOH treatment in hydrogel #1, and these larger K + ions can create more space within the polymer network, allowing for greater water uptake. Additionally, the KOH treatment in hydrogel #2 may result in a higher degree of ionization of the carboxyl groups, leading to increased electrostatic repulsion and greater network expansion. The larger K + ions in hydrogel #2 may also generate a higher osmotic pressure difference between the hydrogel and the surrounding solution, driving more water into the hydrogel structure. Finally, the KOH treatment in hydrogel #2 might induce a more favorable rearrangement of the polymer chains, creating a more open network structure that facilitates greater water absorption. These factors collectively contribute to the higher swelling rate and capacity observed in hydrogel #2 compared to hydrogel #1. The presence of these additional ionized groups in hydrogel #2 creates a more hydrophilic environment within the polymer network, facilitating the penetration and absorption of water. This results in a higher swelling capacity and faster swelling rate compared to the non-treated hydrogel. The enhanced swelling performance of the treated hydrogels, especially treated hydrogel #2, can be highly beneficial for various applications requiring high water absorption and retention capabilities, such as agriculture and personal care products.

**Fig. 3:**
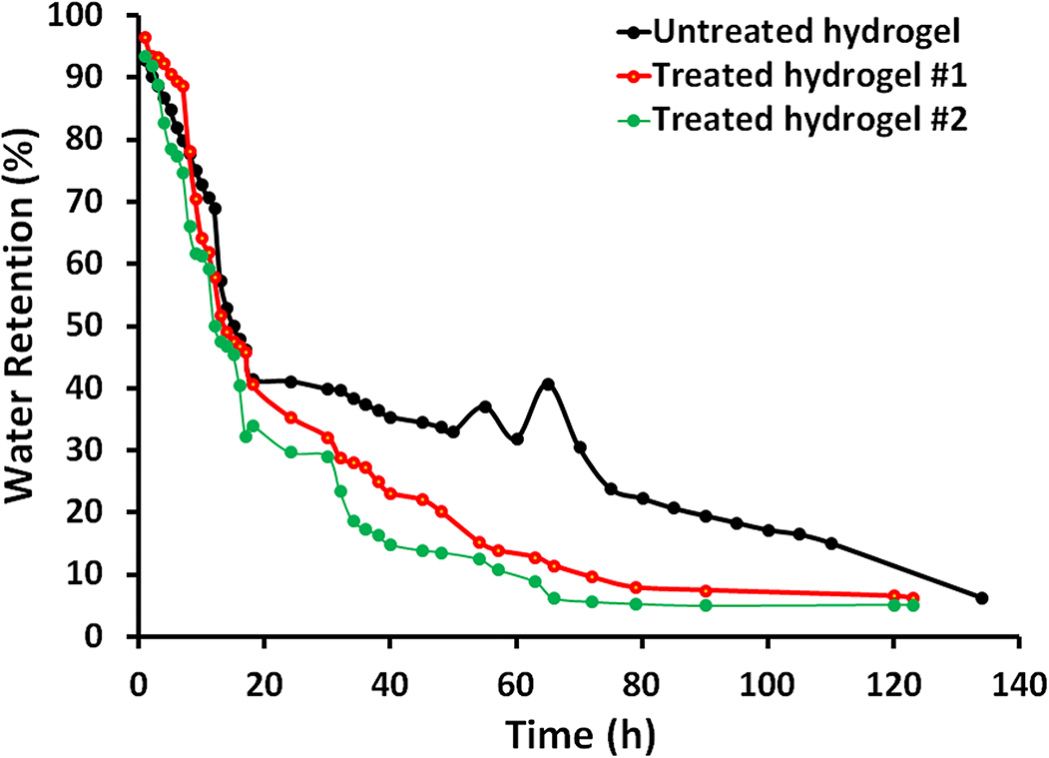
The water retention (%) as a function of time (min) of the prepared untreated hydrogel (black line), treated hydrogel #1 (red line), and treated hydrogel #2 (green line).

Figure 3 shows the water retention (%) as a function of time for the untreated hydrogel, hydrogel #1, and hydrogel #2. Initially, all three hydrogels exhibit high water retention of around 40% in the first 20 h. The data in Fig. 3 shows that the untreated hydrogel (control) initially retains the most water and maintains a higher water retention percentage throughout the observation period, although with some fluctuations between 50 and 80 h. In contrast, the two treated hydrogels (Hydrogel #1 and Hydrogel #2) lose water more rapidly and stabilize at lower retention percentages, with Hydrogel #2 (treated with KOH) exhibiting the most rapid decrease in water retention. A valid point that the treatments applied to the hydrogels, rather than improving water retention, appear to have reduced their water retention capacity compared to the untreated hydrogel. The possible explanation for this observation is that the alkali treatments, such as the NaOH or KOH treatments, may have disrupted the internal structure and network of the hydrogel. The high-water contents in the untreated hydrogel can create internal stresses on the bonds within the hydrogel network, leading to a reduction in water retention. The alkali treatments may have further weakened these bonds, forcing the hydrogel to steadily release water. This resulted in the lower and more rapid water release observed in the treated hydrogels over time. For that, the treated hydrogels can provide good water release within 3–4 days. This strongly depends on the osmotic effect of the soil and the degree of water shortage.

### Scanning electron microscope (SEM) imaging

**Fig. 4:**
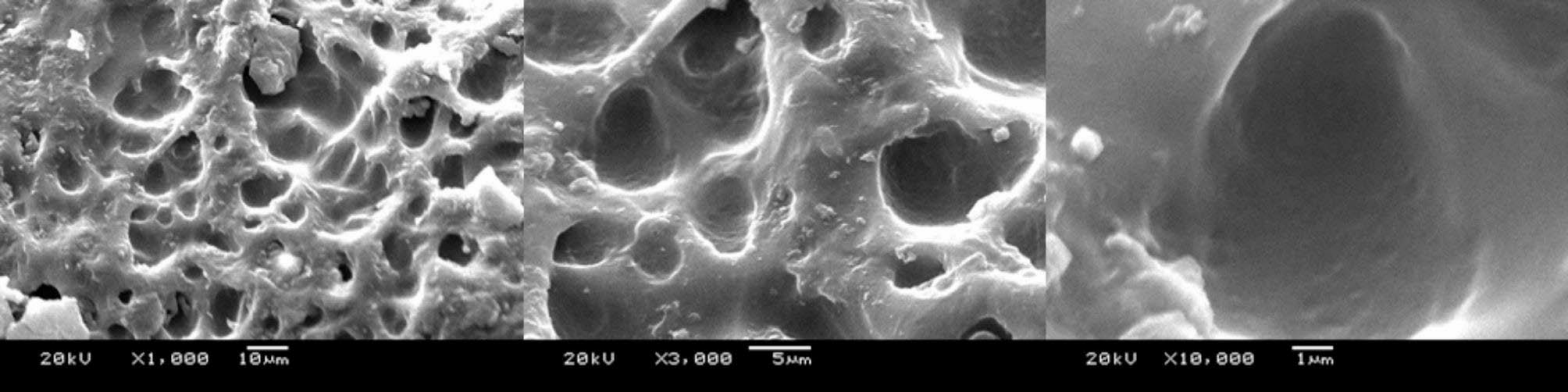
SEM images of the prepared untreated hydrogel at different magnifications (×1000, ×3000, and ×10,000).

**Fig. 5:**
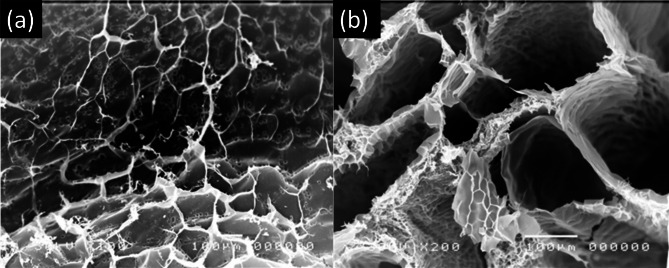
SEM images of the treated hydrogels #2 (×100 and ×200 magnification).

Figure 4 presents scanning electron microscope (SEM) images of the untreated hydrogel at different magnifications (×1000, ×3000, and ×10,000). These SEM images provide valuable insights into the microstructure and morphology of the hydrogel material. At lower magnifications (×1000 and ×3000), the images reveal a highly porous and interconnected structure, which is a desirable feature for superabsorbent hydrogels. The presence of pores and interconnected networks facilitates the rapid absorption and transportation of water molecules throughout the hydrogel matrix. Additionally, the images suggest a relatively rough and irregular surface morphology, which can contribute to increased surface area and enhanced water absorption capabilities. At the highest magnification (×10,000), the SEM image reveals the intricate closed structure of the hydrogel material. Figure 5 presents SEM images of the treated hydrogels #1 and #2 at 100× magnification. These images provide insights into the morphological changes induced by the hydrogel modification treatments with NaOH (hydrogel #1) and KOH (hydrogel #2). Compared to the untreated hydrogel shown in Fig. 4, the hydrogels #1 and #2 exhibit a more uniform and smoother surface morphology. This change in surface topography can be attributed to the alkaline treatment with NaOH and KOH, which likely causes swelling and rearrangement of the polymer chains, resulting in a more compact and homogeneous structure. Additionally, the treated hydrogels appear to have a more interconnected porous network compared to the untreated hydrogel. The formation of these interconnected pores can be a consequence of the alkaline treatment, which may have disrupted the hydrogen bonding interactions within the hydrogel matrix, leading to the formation of new pores and channels. The observed morphological changes, particularly the increased porosity and interconnectivity, can contribute to improved water absorption and retention properties of the treated hydrogels. The interconnected porous structure facilitates the rapid diffusion and transportation of water molecules throughout the hydrogel matrix, while the increased surface area provided by the pores enhances the overall water-binding capacity. It is worth noting that the extent of morphological changes may vary between hydrogel #1 and hydrogel #2, as the treatment could lead to differences in the degree of polymer chain swelling, rearrangement, and subsequent pore formation. The size of the porosity is increased for hydrogel #2 than hydrogel #1.

### The fertilizer release behavior of (urea, K and P) from hydrogel #2

**Fig. 6:**
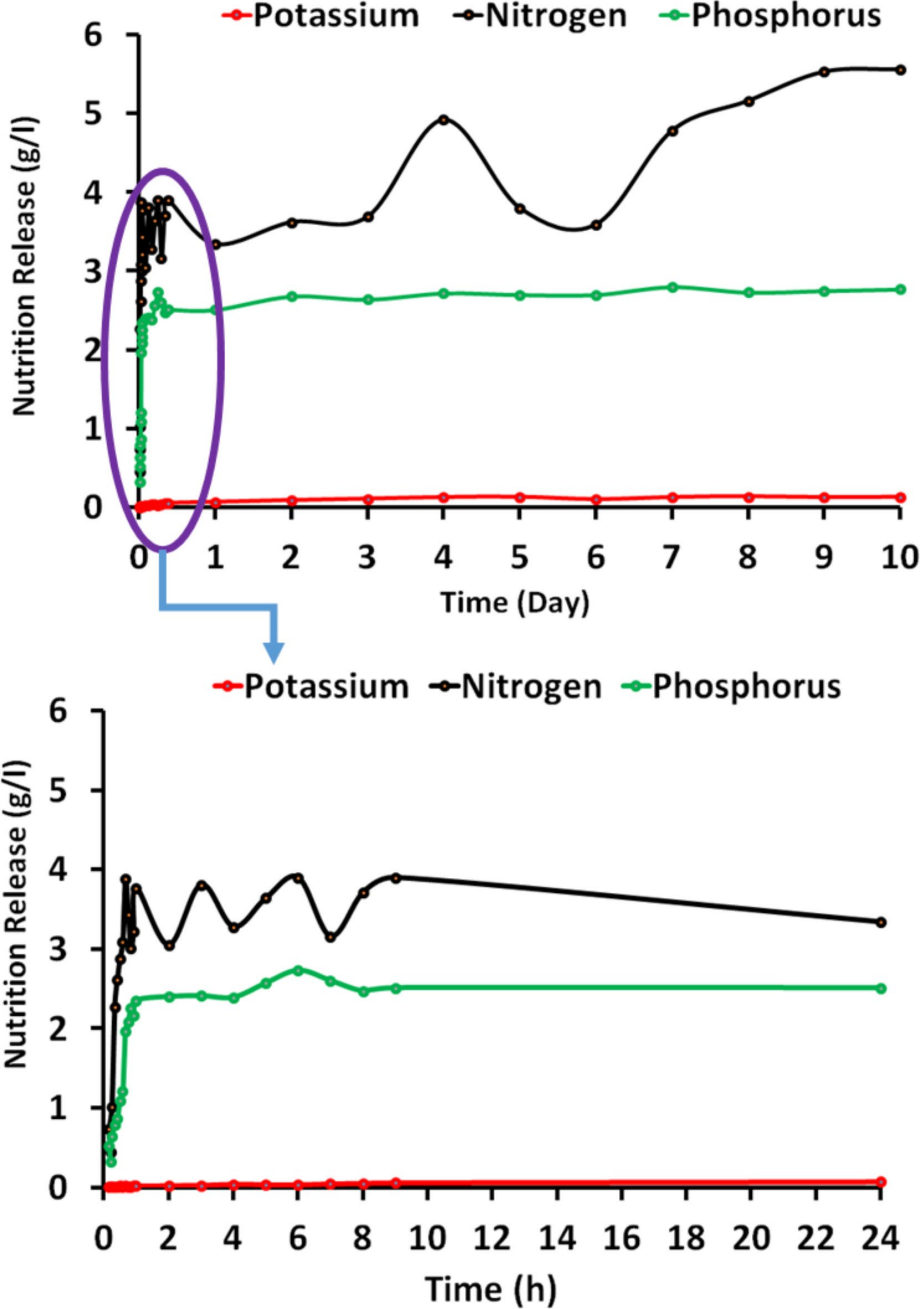
Three fertilizer release as function of time (from 5 min to 10 days) using hydrogel #2.

Figure 6 presents the release profiles of three essential fertilizers—potassium (K), nitrogen (N), and phosphorus (P) - from the hydrogel #2 over an extended period of 10 days. The data shown in Fig. 6 corresponds to the values plotted in the graph, providing quantitative information on the release kinetics of these nutrients. The release of potassium from the hydrogel #2 exhibits a distinct burst release phase followed by a sustained and gradual release over time. Initially, within the first 5 min, a rapid release of 23.84 mg/L (2.38%) of potassium is observed. This burst release can be attributed to the potassium ions present near the surface or loosely bound regions of the hydrogel matrix, which are readily released upon contact with the external aqueous medium. As time progresses, the release rate slows down, indicating a transition from the burst release phase to a diffusion-controlled release mechanism. At 1 h, the potassium release concentration reaches 17 mg/L (1.7%), and it continues to increase gradually, reaching 58 mg/L (5.8%) at 9 h, and 70 mg/L (7%) at 1 day. Beyond the initial burst release phase, the potassium release follows a relatively linear trend, with concentrations reaching 130 mg/L (13%) at 5 days and 136 mg/L (13.6%) at 8 days. This sustained release behavior can be attributed to the diffusion of potassium ions from the intricate hydrogel network to the external solution, driven by concentration gradients. It is noteworthy that the release rate appears to plateau towards the end of the 10-day period, with a maximum concentration of 129 mg/L (12.9%) observed at 10 days. This plateau suggests a gradual depletion of the potassium reserves within the hydrogel matrix, as the concentration gradient between the hydrogel and the external solution diminishes over time. The release profile for nitrogen exhibits a more gradual and sustained release pattern compared to potassium. The initial burst release phase is less prominent, with a concentration of 45 mg/dL (0.45 g/L) observed at 5 min. As time progresses, the nitrogen release rate increases steadily, reaching concentrations of 227 mg/dL (2.27 g/L) at 4 h, 388 mg/dL (3.88 g/L) at 7 h, and 553 mg/dL (5.53 g/L) at 10 days. The relatively linear release pattern observed for nitrogen suggests a diffusion-controlled release mechanism, wherein the nitrogen species must traverse through the intricate hydrogel network to reach the external solution. It is worth noting that the nitrogen release data exhibits some fluctuations, with occasional decreases in concentration observed at certain time points (e.g., 50 h and 192 h). These fluctuations could potentially arise from experimental variations or analytical uncertainties during the measurement process. Similar to nitrogen, the release profile for phosphorus from the hydrogel #2 exhibits a gradual and sustained release behavior. The initial burst release phase is less pronounced, with a concentration of 33 mg/dL (0.33 g/L) observed at 5 min. As time progresses, the phosphorus release rate increases steadily, reaching concentrations of 79 mg/dL (0.79 g/L) at 4 h, 197 mg/dL (1.97 g/L) at 7 h, and 277 mg/dL (2.77 g/L) at 10 days. Like nitrogen, the relatively linear release pattern observed for phosphorus suggests a diffusion-controlled release mechanism governed by concentration gradients within the hydrogel matrix. It is noteworthy that the release rates for both nitrogen and phosphorus appear to be influenced by the concentration gradients established within the hydrogel, leading to a sustained release pattern over the 10-day period. This sustained release behavior can be highly beneficial in agricultural applications, as it allows for a controlled and prolonged delivery of essential nutrients to the soil, minimizing the risk of nutrient leaching and promoting efficient utilization by plants. Overall, the hydrogel #2 exhibits tailored release profiles for potassium, nitrogen, and phosphorus, with an initial burst release phase followed by a sustained and gradual release over an extended period. The sustained release behavior can be attributed to the diffusion-controlled release mechanism governed by the intricate hydrogel network and concentration gradients. These release characteristics make the hydrogel #2 a promising material for applications in agriculture, where controlled and prolonged delivery of essential nutrients is crucial for optimal plant growth and nutrient utilization.

The fomented analyses support our proposal of hydrogel formation mechanism. The formation reaction of the present hydrogel is initiated by the generation of free-radical initiators using mixed persulfate; the free radicals are formed on the PVP backbone, leading to the activation of the vinyl pyrrolidone groups. The activated vinyl pyrrolidone groups on the PVP backbone undergo grafting reactions with acrylic acid (AA) monomers. This results in the formation of PVP-g-PAA copolymer chains, where the PAA (polyacrylic acid) moieties are grafted onto the PVP backbone. Crosslinking agents; N,N′-methylenebisacrylamide (MBA), is incorporated into the reaction mixture. The crosslinking agents form covalent bonds between the PVP-g-PAA copolymer chains, creating a three-dimensional network structure. The crosslinking process stabilizes the hydrogel structure with Arabic gum and enhances its swelling and water retention properties. The as-synthesized PVP-based SAH is further alkali treated with either NaOH or KOH. The alkali treatment leads to the partial neutralization of the carboxylic acid groups (-COOH) in the PAA moieties, converting them to carboxylate groups (-COO⁻). This increases the hydrophilicity and swelling capacity of the SAH by introducing additional ionic groups.

### Investigating the impact of water stress on the morphological features and yield of *Pisum sativum* with the application of superabsorbent hydrogel #2

**Fig. 7:**
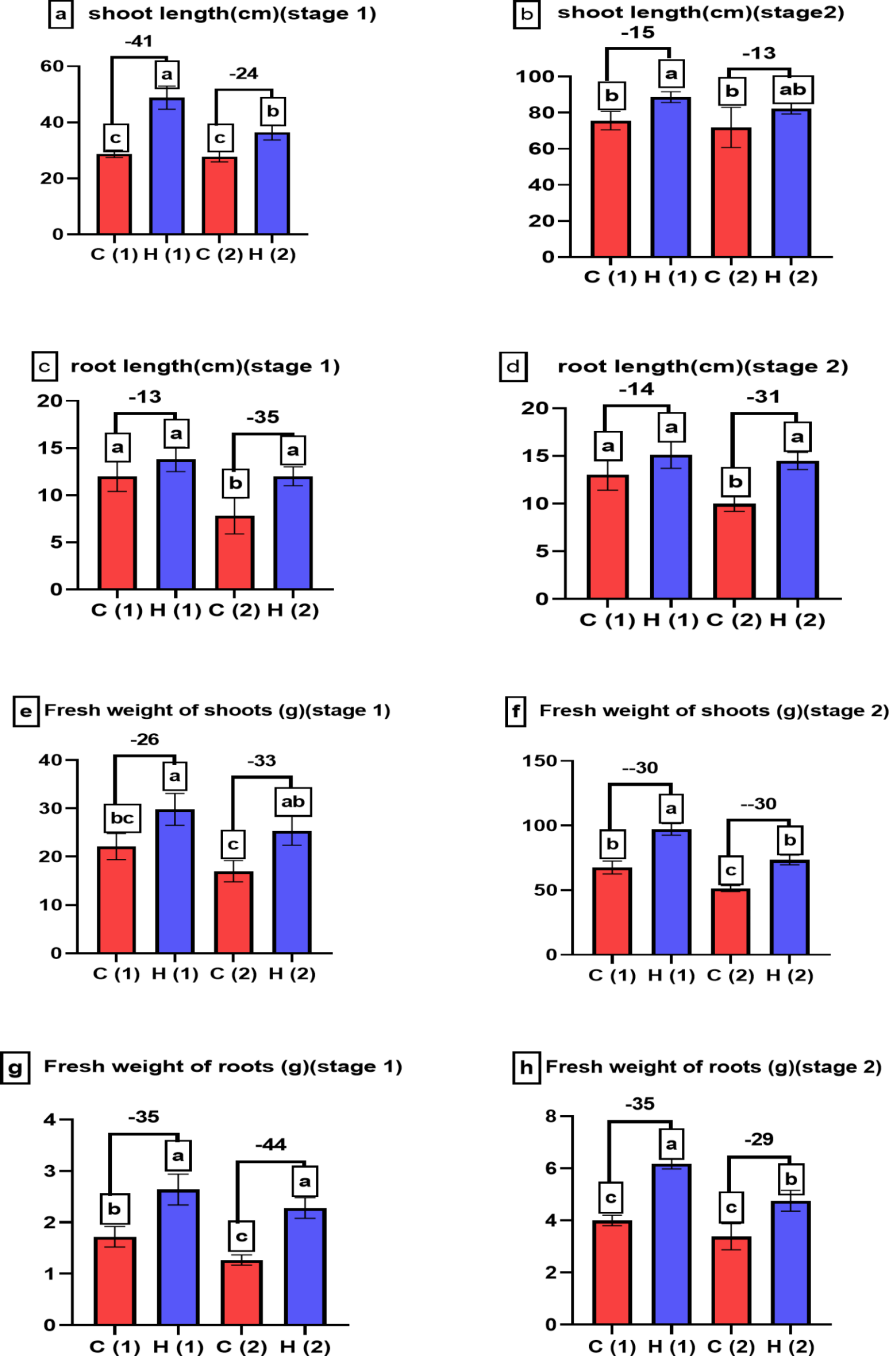
Explains how varying levels of water drought stress (0% and 100%) affect the morphological characteristics of *Pisum sativum* at two growth stages. Parameters assessed include (**a**,**b**) shoot length (in cm), (**c**,**d**) root length (in cm), (**e**,**f**) fresh weight of shoots (in grams), and (**g**,**h**) fresh weight of roots (in grams). The treatments’ significant differences (*P* < 0.05) are shown by the alphabets.

**Fig. 8:**
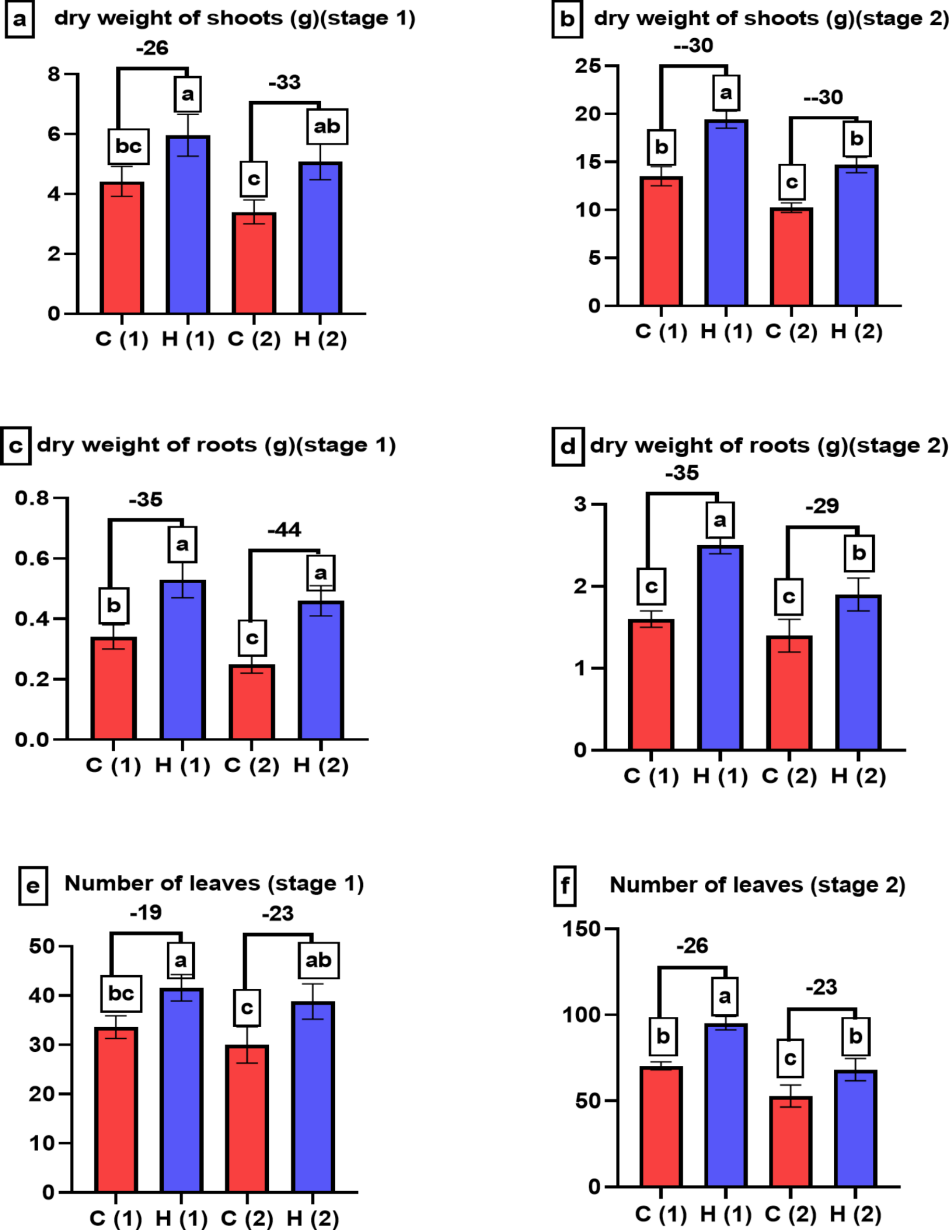
Shows how varying levels of water drought stress (0% and 100%) affect the morphological characteristics of *Pisum sativum* at two growth stages. Parameters assessed include (**a**,**b**) dry weight of shoots (in grams), (**c**,**d**) dry weight of roots (in grams) and (**e**,**f**) number of leaves. The treatments’ significant differences (*P* < 0.05) are shown by the alphabets.

Figures 7 and 8 in the study investigated the effects of varying water drought stress levels on the morphological characteristics of *Pisum sativum* at two growth stages, with or without the application of superabsorbent hydrogel (SAH). Specifically, Fig. 7 focuses on shoot length, root length, and the fresh weight of both shoots and roots. Under 100% water stress, shoot length increased by 24% and 13% at stages 1 and 2, respectively, while root length increased by 35% and 31% in the same stages compared to control. Figure 8 examines the dry weight of shoots and roots, as well as the number of leaves, showing similar positive effects with SAH treatment: dry weight of shoots and roots increased significantly, and the number of leaves was enhanced by 23% at both stages. These findings suggest that SAH effectively mitigates the adverse effects of drought stress on *Pisum sativum*, improving both growth and biomass accumulation.

**Fig. 9:**
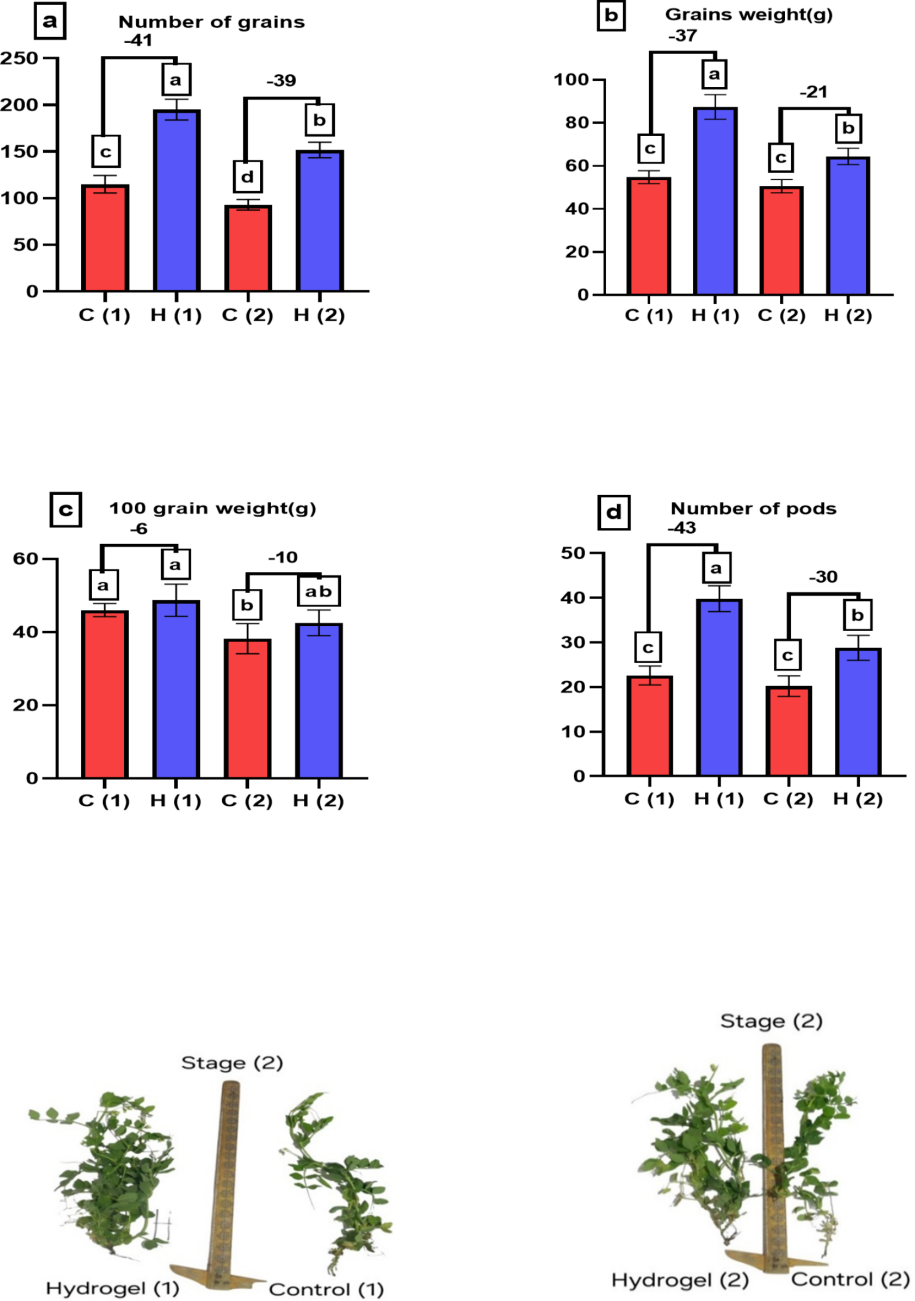
Portrays how varying levels of water drought stress (0% and 100%) affect the yield parameter of *Pisum sativum*. Parameters assessed include (**a**) the number of grains, (**b**) grain weight (in grams), (**c**) 100 grain weight (in grams), and (**d**) the number of pods. The treatments’ significant differences (*P* < 0.05) are shown by the alphabets.

Figure 9 of the study evaluates the yield parameters of *Pisum sativum* under different levels of drought stress, with and without the application of SAH. Figure 9a shows that the number of grains increases markedly with SAH, demonstrating the hydrogel’s role in enhancing reproductive output even under severe water stress. These results in parallel with our previous study on another plant and hydrogel^17^ which stated that, the yield parameters of *Phaseolus vulgaris* showed a notable improvement in presence hydrogel (Polyacrylic Acid (PAAc)/carboxymethyl cellulose (CMC)). The vital role of hydrogel may be as a result of numerous reasons, including smaller particle sizes and increased surface area, can be responsible for this, since it leads to hydrogels absorbing water more forcefully. Figure 9b highlights an increase in the total grain weight, indicating better grain filling and development. Figure 9c presents a higher 100-grain weight, which reflects the improved grain quality and size. Finally, Fig. 9e illustrates an increase in the number of pods, suggesting that SAH helps maintain overall plant productivity by ensuring more pods develop and mature properly. These results underscore the efficacy of SAH in mitigating the adverse effects of drought stress, thereby enhancing both the quantity and quality of the yield in *Pisum sativum*. In both stages, the application of SAH under 100% water stress significantly improves yield outcomes.

### Investigating the impact of different degrees of water stress on pigment and carotenoid content of *Pisum sativum* with the application of superabsorbent hydrogel #2

**Fig. 10:**
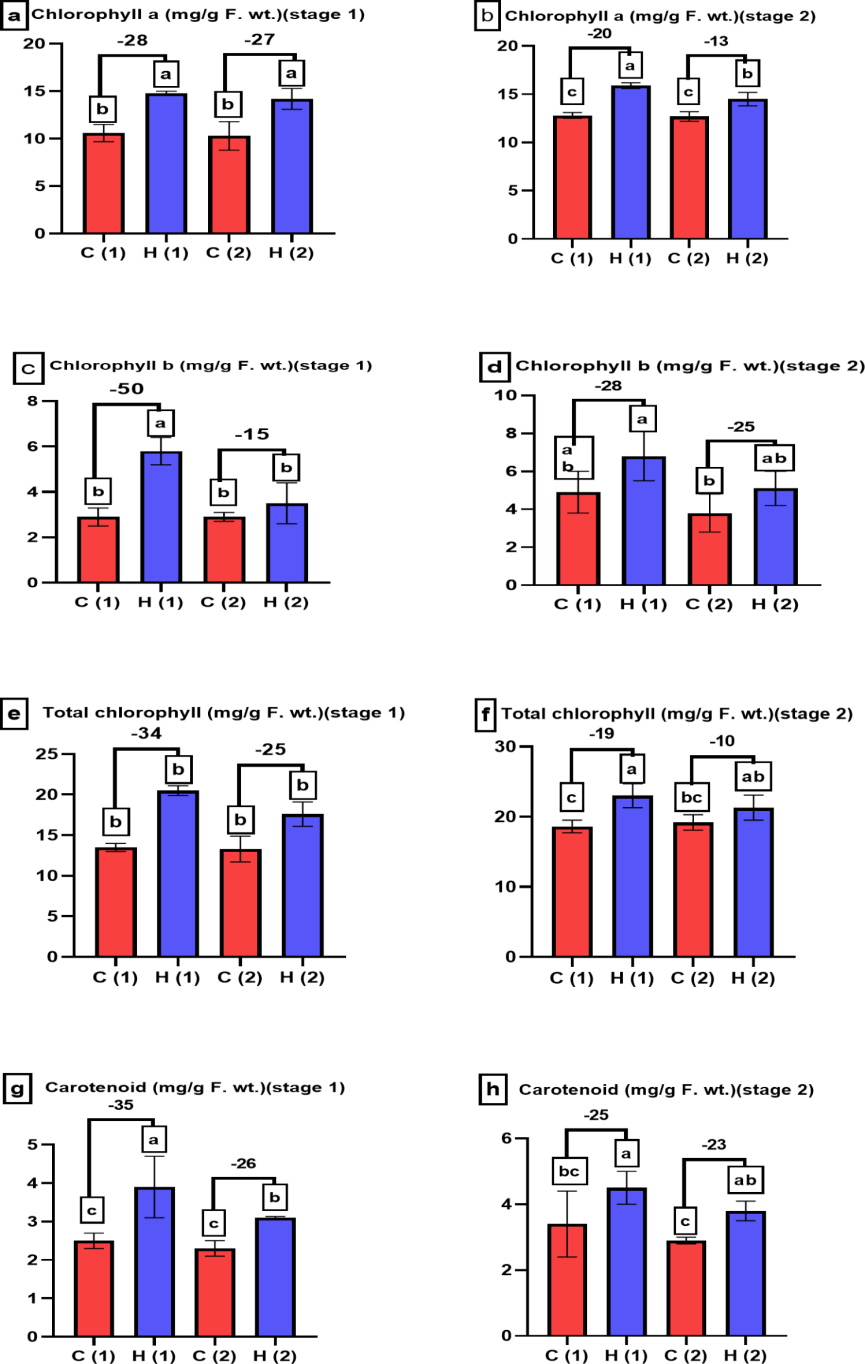
Examines how varying levels of water drought stress (0% and 100%) affect the pigment content of *Pisum sativum* at two growth stages. Parameters assessed include (**a**,**b**) chlorophyll a, (**c**,**d**) chlorophyll b, (**e**,**f**) total chlorophyll and (**g**,**h**) carotenoids. The treatments’ significant differences (*P* < 0.05) are shown by the alphabets.

Chlorophyll is a green pigment found in the chloroplasts of plants, algae, and some bacteria. It plays an important role in photosynthesis, hydrogel enhanced chlorophyll a in Fig. 10a, b, chlorophyll b in Fig. 10c, d, total chlorophyll in Fig. 10e, f and carotenoid content in Fig. 10g, h in *Pisum sativum* either under normal condition or under drought stress condition, the results were compatible with our previous research on sunflower^18^.

### Investigating the impact of different degrees of water stress on stress indicators (anti-oxidant enzymes, H_2_O_2_ and MDA) of *Pisum sativum* with the application of superabsorbent hydrogel #2

**Fig. 11:**
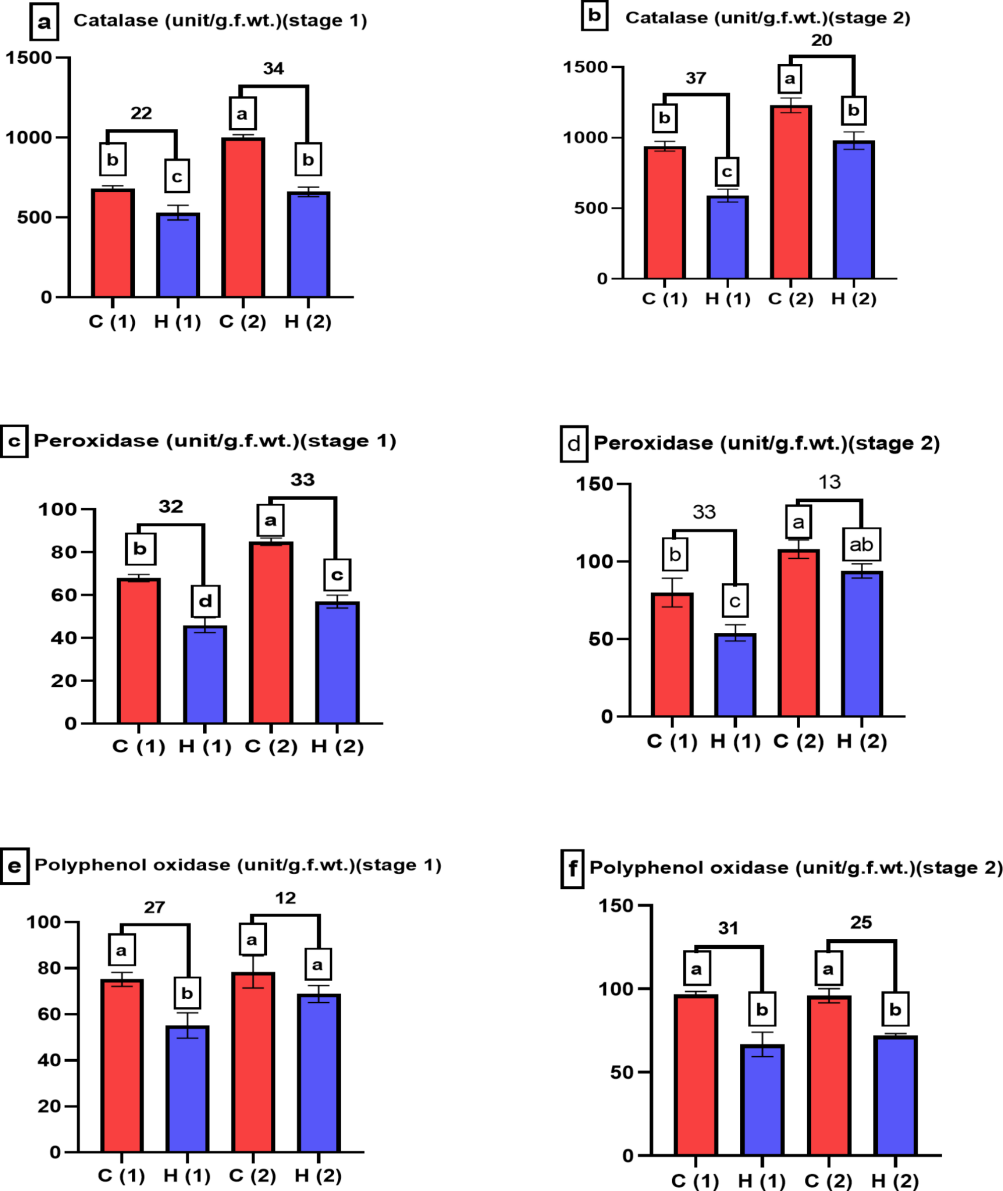
Examines how varying levels of water drought stress (0% and 100%) affect the anti-oxidant enzymes of *Pisum sativum* at two growth stages. Parameters assessed include (**a**,**b**) catalase, (**c**,**d**) peroxidase, and (**e**,**f**) polyphenol oxidase. The treatments’ significant differences (*P* < 0.05) are shown by the alphabets.

**Fig. 12:**
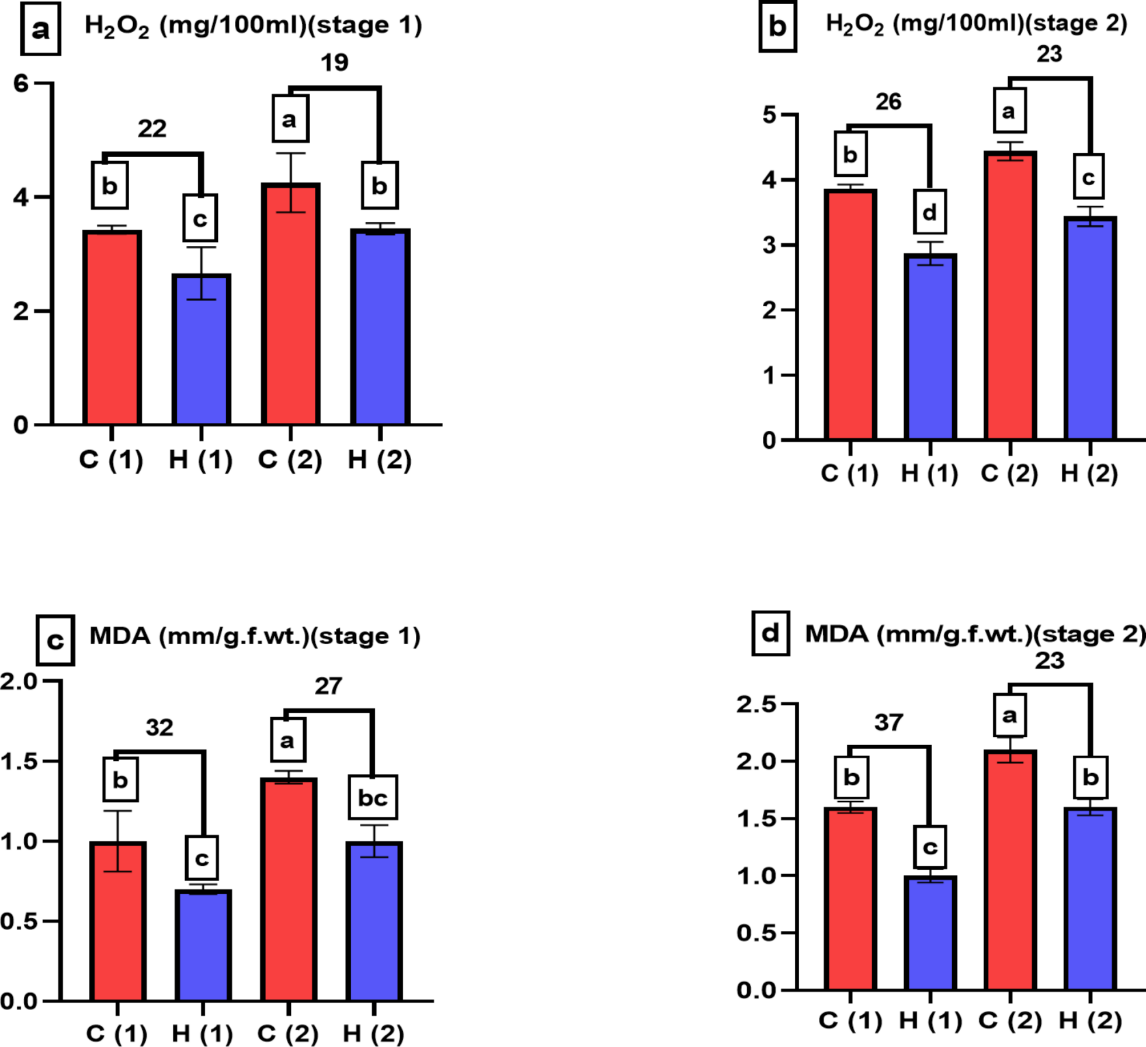
Shows how varying levels of water stress (0% and 100%) affect H_2_O_2_ and MDA of *Pisum sativum* at two growth stages. Parameters assessed include (**a**,**b**) H_2_O_2_ (in mg/100 ml) and (**c**,**d**) MDA (in mg/g.f.wt). The treatments’ significant differences (*P* < 0.05) are shown by the alphabets.

Plants have developed various defense mechanisms to cope with oxidative stress, which can arise from factors such as exposure to drought, Antioxidant enzymes are a key component of these defense mechanisms, in response to water stress, plants had an increase in antioxidant enzyme activity^27^. The decrease of anti-oxidant enzymes associated to hydrogel treatments indicated that hydrogel decreased the drought stress, catalases decreased by 34% and 20% at stage 1 and stage 2 respectively at 100% stress as presented in Fig. 11a, b, in the same contest^28^ proved that hydrogel decreased catalase enzymes of basil plants. Also peroxidases decreased by 33% and 13% as presented in Fig. 11c, d, these results agree with^29^ on another hydrogel and another plant, who proved that hydrogel (Potassium poly acrylate poly acrylamide) decreased peroxidase enzyme of *Triticum aestivum plants*, also polyphenol oxidases decreased by 12% and 25% as presented in Fig. 11e, f, in the same line we found that SAH decreased polyphenol oxidase in maize plant under drought condition^30^, H_2_O_2_ plays diverse and essential roles in plant physiology, ranging from signaling and defense to growth regulation and stress responses, the increase in H_2_O_2_ content indicate the response of plant to stress, that concept in agreement with our result presented in Fig. 12a, b, hydrogel help plant to decrease stress as well as decrease H_2_O_2_ content, it agree with our previous results in *Zea maize*^31^, To offset the oxidative stress brought on by the water shortage, the plant’s cells release H_2_O_2_ when water stress occurs. Plants’ ability to produce H_2_O_2_ can be reduced by the hydrogel by lessening water stress. Under drought stress a plant produces more reactive oxygen species (ROS) than it can detoxify so, oxidative stress results. Plant cells may be harmed by this, leading to the production of malondialdehyde (MDA), which is a sign of oxidative damage. Malondialdehyde (MDA) is a marker commonly used to assess lipid peroxidation in plants, which is a result of oxidative stress, when plants are exposed to various environmental drought, data presented in Fig. 12c, d indicated that SAH decreased MDA content under drought condition, agreed with our results^32^, which revealed that the positive effect of the used superabsorbent hydrogel #2 was proved by lower concentration of malonaldehyde (MDA)as well as mitigation the water stress by enhancing plant water uptake.

### Investigating the impact of different degrees of water stress on shoot and seed analysis of *Pisum sativum* with the application of superabsorbent hydrogel #2

**Fig. 13:**
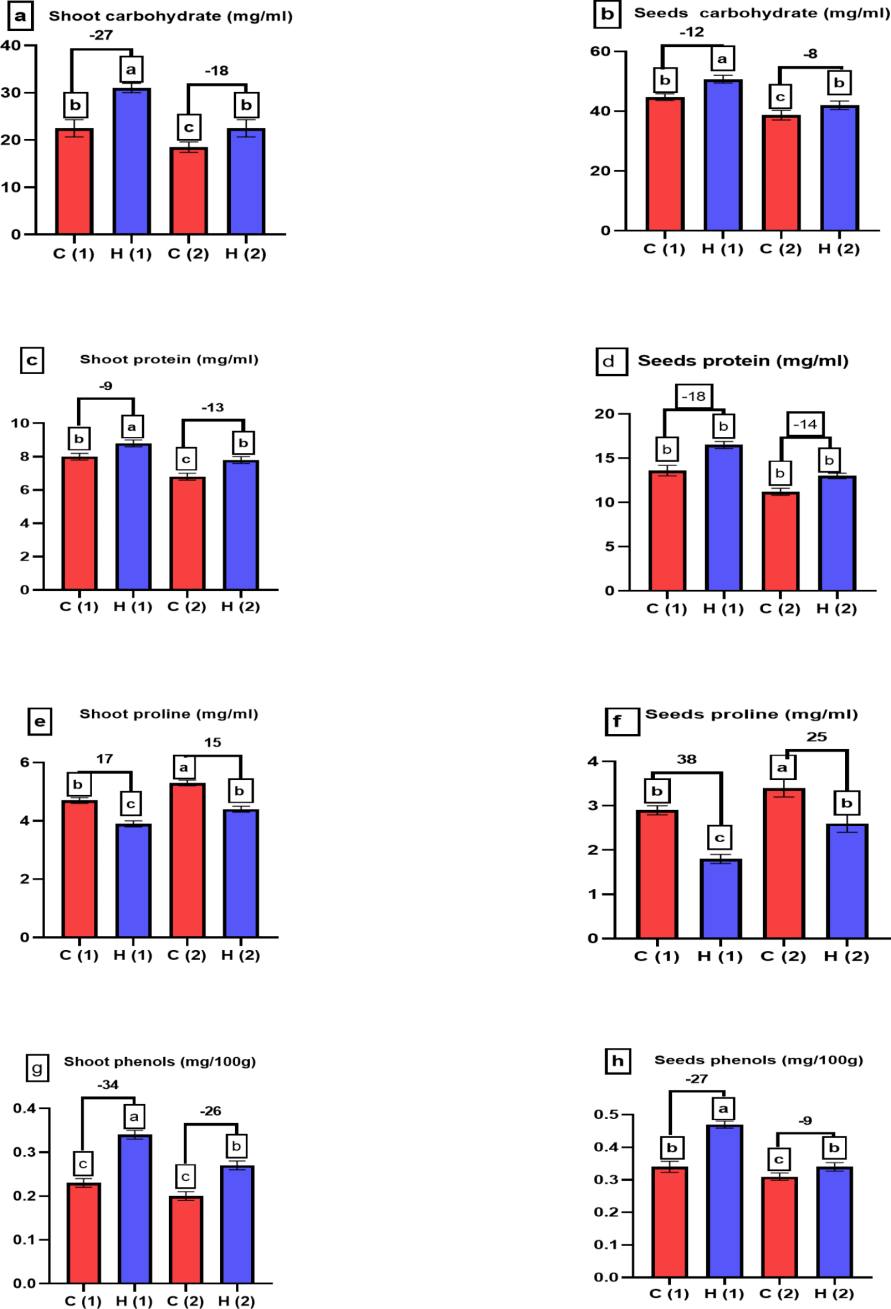
Examines how varying levels of water drought stress (0% and 100%) affect the shoot and seed analysis of *Pisum sativum* at two growth stages. Parameters assessed include (**a**,**b**) carbohydrate content (in mg/ml), (**c**,**d**) protein content (in mg/ml), (**e**,**f**) proline content (in mg/ml) and (**g**,**h**) phenol content (in mg/100 g). The treatments’ significant differences (*P* < 0.05) are shown by the alphabets.

Data presented in Fig. 13a, b showed the positive effect of SAH that enhanced carbohydrate content in plant by 18% and 8% at two stages respectively, also there are enhancement in protein content Fig. 13c, d, these results agree with^33^ who illustrated the positive effect of hydrogel on carbohydrate and protein content of Brazilian Cerrado plants. Proline is an important amino acid found in plants, playing various roles in stress responses, Regarding proline, our data in Fig. 13e, f showed that SAH decreased shoot and seed proline content by 16% and 26% at 100% stress respectively, in the same line^34^ found that using of hydrogel decreased proline content as compared to control. Also^33^ showed the vital role of hydrogel (dextrin /polyacrylamide) in decrease proline content of sunflower plants under drought stress which act hydrogel decrease the harmful effect of drought stress. Phenolic compounds are secondary metabolites found in plants; phenolic compounds are potent antioxidants, capable of scavenging free radicals and reducing oxidative stress in plants. They protect plant cells from damage caused by reactive oxygen species (ROS), SAH increased phenolic compound in *Pisum sativum*, Fig. 13g, h this results agreed with our previous research on *Zea maize*^33^. These findings in our study support the vital function of SAH in mitigating the detrimental effects of drought stress on *Pisum sativum* plants, which hydrogel has the large capacity to increase nutrient retention, decrease irrigation, and boost crop yield. Pumping water from its source to the crops using less irrigation water uses less energy. When employing hydrogels, combined decreases in the volume of irrigation water and fertilizer applied could offer significant advantages and cost savings for farming operations.

## Conclusions

The presented study successfully developed a multifunctional PVP-based superabsorbent hydrogel (SAH) capable of controlled fertilizer release and enhanced water retention in soil. The treated hydrogels demonstrated exceptional swelling capacities and water retention properties, attributed to their porous and interconnected structure. The controlled release profiles revealed an initial burst release followed by a sustained and gradual release of essential fertilizers (nitrogen, phosphorus, and potassium) over an extended period, enabling prolonged nutrient delivery to plants. The application of SAH effectively mitigated the detrimental effects of drought stress on *Pisum sativum*, demonstrating its potential in sustainable agriculture by improving various morphological traits, including shoot length, root length, fresh and dry weights, leaf number and yield parameters under water stress conditions. Furthermore, hydrogel enhanced reproductive output by increasing yield parameters, such as grain number, grain weight, 100-grain weight, pod number. Also, it improves grain quality by increasing nutritional value of grains (protein, carbohydrate and phenol contents) compared to untreated plants. The hydrogel also exhibited a protective role against oxidative stress, as evidenced by reduced levels of hydrogen peroxide and malondialdehyde, markers of oxidative damage. Additionally, it enhanced the levels of chlorophyll and carotenoid pigments, crucial for photosynthesis and plant productivity.

The unique contribution of our work lies in the development of a multifunctional PVP-based superabsorbent hydrogel that combines cost-effectiveness, reliability, and enhanced performance. Unlike existing solutions, the present hydrogel demonstrates several advantages including (1) superior water retention in which maintaining over 60% water retention even after 10 days, significantly outperforming conventional hydrogels. (2) Controlled fertilizer release in which our hydrogel exhibits a tailored release profile for essential nutrients (N, P, K), providing sustained nutrient delivery over an extended period. (3) Enhanced plant performance in which application of this hydrogel mitigated the adverse effects of drought stress on *Pisum sativum*, improving morphological traits, yield parameters, and antioxidant responses. (4) Cost-effective synthesis in which the use of readily available materials and a straightforward synthesis process makes our hydrogel economically viable for large-scale agricultural applications. (5) Versatility in which the ability to modify the hydrogel properties through simple alkaline treatments (NaOH or KOH) offers flexibility in tailoring the hydrogel for specific agricultural needs. These features collectively represent a significant advance over existing hydrogel solutions in terms of performance, reliability, and potential for practical implementation in sustainable agriculture.

While the results of this study demonstrate the promising potential of the PVP-based superabsorbent hydrogel (SAH) for controlled fertilizer release and enhanced water retention in *Pisum sativum* under drought stress, some limitations should be acknowledged. While the PVP-based SAH is designed to be biodegradable, its long-term fate and potential environmental impact in agricultural ecosystems need to be thoroughly investigated, especially concerning non-target organisms and soil microbiomes. We recommended exploring another eco-friendly multifunctional hydrogel that has more water retention, has the gradual release of another essential fertilizer (magnesium, calcium, iron, etc.), and acts as a carrier to many anti-phytopathogenic agents. Moreover, the current study focused on *Pisum sativum* as the model crop. Evaluating the performance of the SAH across a wider range of economically important crop species and cultivars would demonstrate the broader applicability of this technology.

## Electronic supplementary material

Below is the link to the electronic supplementary material.

Supplementary Material 1

## Data Availability

All data generated or analysed during this study are included in this published article or supplementary information file.
