# Supplementary material for: Synthesis and application of a multifunctional poly (vinyl pyrrolidone)-based superabsorbent hydrogel for controlled fertilizer release and enhanced water retention in drought-stressed Pisum sativum plants
Source: Sci Rep. 2024 Nov 12;14:27734. doi: 10.1038/s41598-024-76255-7 (PMC11557843; doi:10.1038/s41598-024-76255-7)
Supplement: Supplementary file 1 — Supplementary Material 1 [file 41598_2024_76255_MOESM1_ESM.docx]

**Supplementary File**

**Synthesis and application of a multifunctional poly (vinyl pyrrolidone)-based superabsorbent hydrogel for controlled fertilizer release and enhanced** **water retention in drought-stressed** ***pisum sativum* plants**

Mohamed Mohamady Ghobashy^1^, Mohamed A. Amin^2^, Abeer E. Mustafa^3^, Mahmoud A. El-diehy^2^, Basem Kh. El‑Damhougy^4^, Norhan Nady ^5, *^

^1^ Radiation Research of Polymer Department, National Center for Radiation Research and Technology (NCRRT), Atomic Energy Authority, Nasr City, P.O. Box 29, Cairo, Egypt. Mohamed.ghobashy@eaea.org.eg

^2^ Department of Botany and Microbiology, Faculty of Science, Al-Azhar University, Cairo 11884, Egypt. [Mamin7780@yahoo.com](mailto:Mamin7780@yahoo.com); docmahmoud7@gmail

^3^ Department of Botany and Microbiology, Faculty of Science (girls), Al-Azhar University, Nasr City, Cairo 11884, Egypt. Abbermostafa715.el@azhar.edu.eg

^4^ Department of Chemistry, Faculty of Science, Al-Azhar University, Cairo 11884, Egypt. basemkh88@yahoo.com

^5^ Polymeric Materials Research Department, Advanced Technology and New Materials Research Institute (ATNMRI), City of Scientific Research and Technological Applications (SRTA-city), New Borg El-Arab City, Alexandria 21934, Egypt.

⁎ Corresponding author. E-mail address: [norhan.nady77@yahoo.com](mailto:norhan.nady77@yahoo.com), Tel: +201090918521

[**Table 1**](#Table_1)**s.** Physiochemical estimation of the soil.

| **Soil Texture** | | **Sand (%) > 200 - 20 μm** | | | **Silt (%) 20 - 2 μm** | | | **Clay (%) < 2 μm** | |
| --- | --- | --- | --- | --- | --- | --- | --- | --- | --- |
| Sandy loam | | 65.72 | | | 20.00 | | | 14.28 | |
| **CaCO3**  **%** | **Cations meq / l** | | | | **Anions meq / l** | | | **ECe**  **(dS/m)** | **PH at**  **1:2.5** |
| 4.20 | **K^+^** | **Na^+^** | **Mg^++^** | **Ca^++^** | **Cl^-^** | **HCO_3_^-^** | **CO_3_^--^** | 1.80 | 7.47 |
|  | 0.36 | 7.89 | 3.25 | 4.90 | 8.05 | 4.65 | * |  |  |
| **Conc. (mg/kg soil)** | | | | | | |  |  |  |
| **N** | **K** | **P** | **Cu** | **Fe** | **Mn** | **Zn** |  |  |  |
| 32.44 | 205.40 | 14.60 | 4.82 | 16.00 | 0.71 | 4.05 |  |  |  |

| 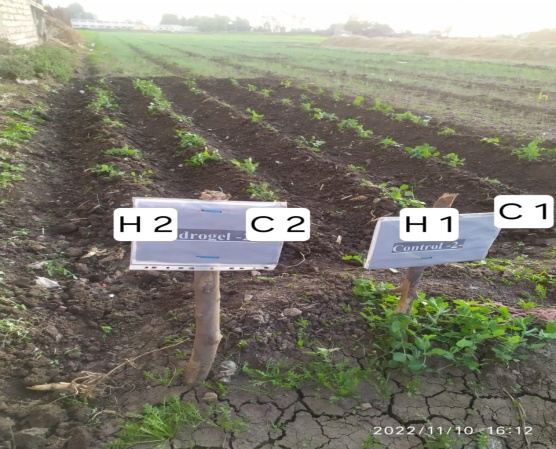 |
| --- |
| [**Figure 1**](#Table_1)**s.** Utilization of SAH on *Pisum sativum* at Various Stress Levels (0% (1) and 100% (2). The control samples (without hydrogel) are denoted by the letter (C), while the hydrogel-enhanced samples are represented by the letter (H). |

[**Table 2**](#Table_1)**s.** Key words of treatments

| **Irrigation Time (days)** | **Control** | **Hydrogel** | **Water stress (%)** |
| --- | --- | --- | --- |
| 10 | C1 | H1 | 0 |
| 20 | C2 | H2 | 100 |

1. **Pigment and Carotenoid Identification**

In 1966, fresh green leaves weighing one gram were processed according to the technique outlined by **[1]**. The method involved finely mincing the leaves, followed by pigment extraction through the grinding of leaves pieces in a solution consisting of 100 ml of 80% acetone. Subsequently, the resulting mixture underwent filtration, and the filtrate was meticulously transferred to a 100 ml volumetric flask, adjusting the volume to 100 ml with 80% acetone. Optical density measurements were taken at specific wavelengths, namely 649 nm and 665 nm. The quantities of chlorophylls a and b, as well as their total amounts in plant tissues, can be computed using the following equations:

Chlorophyll a (Mg/g tissue) = 11.63 (A665) - 2.39 (A649)

Chlorophyll b (Mg/g leaves) = 20.11 (A649) - 5.18 (A665)

Total chlorophyll a + b (Mg/g tissue) = 6.45 (A665) + 17.72 (A649)

For the estimation of carotenoid chemical composition, the method described by **[2]** is applicable:

Carotenoids (mg/g fresh weight) = (1000 * A470) – (1.82 * chlorophyll a) - (85.02 * chlorophyll b) / 198.

Note that "(A)" denotes the optical density in these calculations.

1. **Estimation of** **total phenolic compounds**

Using the method outlined in [3], the amount of phenolics present in the dried root and shoot tissues of ***Pisum sativum*** plants was ascertained. For 24 h., one gram of plant tissue was immersed in 80% ethanol (5–10 mL). After filtering the mixture, the leftover material was extracted twice more using the same solvent. Each extract was mixed together with ethanol to make a final amount of 50 milliliters. For three minutes, the 0.5 mL of extract and the 0.5 mL of Folin reagent were thoroughly combined and agitated. Next, 1 mL of saturated sodium carbonate solution and 3 mL of distilled water were added, and thoroughly mixed. The absorbance was measured spectrophotometrically at 725 nm after one hour.

1. **Determining** **Free Proline Levels in Plant Tissues:**

In **[4]** Procedure:

- 1. Grind 0.5 g of dried tissues and combine with 10 ml of 3% sulfosalicylic acid.
  2. Filter the resulting mixture to obtain the extract.
  3. In a test tube, mix 2 ml of the extract, 2 ml of acid ninhydrin, and 2 ml of glacial acetic acid.
  4. Heat the mixture for 1 hour, and then terminate the process in an ice bath.
  5. Extract the tube contents with 4 ml of toluene, vigorously mixing and separating the top layer.
  6. Measure the color absorbance at 520 nm using a UV-colorimeter, with toluene as a blank.
  7. Construct a standard curve using known proline concentrations to determine levels based on sample dry weight, utilizing the following equation:

| Mg/g proline = | (X) PPM * ml Extract volume | |
| --- | --- | --- |
|  | 2 * Sample dry weight * 100 |  |

1. **Extraction and Quantification of Catalase, Peroxidase, and Polyphenol Oxidase Enzymes**

- **Extraction:**

For the measurement of antioxidant enzymes (catalase, peroxidase, and polyphenol oxidase), terminal shoots and young leaves were utilized. The extraction process involved digesting 2 g of plant pellets with 10 ml of pH 6.8 phosphate buffer. Subsequently, the mixture underwent centrifugation at 20,000 rpm for 20 min at 20°C, yielding a clear supernatant containing the enzymes. This supernatant served as the source of enzymes for further analysis **[5].**

- **Calculation of Catalase Activity:**

The reaction mixture, totaling 10 ml, consists of 40 µl of enzyme extract and 9.96 ml of a phosphate buffer solution at pH 7.0 containing oxygenated water. Oxygenated water is prepared by adding 0.16 ml of 30% hydrogen peroxide to 100 ml of 50 mM phosphate buffer. Catalase activity is determined by measuring the change in H2O2 absorbance at 250 nm over 60 seconds using a UV-colorimeter. To establish a blank, the enzyme extract is replaced with buffer solution. In summary, catalase activity is assessed by monitoring the reduction in H_2_O_2_ absorbance over 60 seconds in a reaction mixture containing enzyme extract, phosphate buffer, and hydrogen peroxide, while a blank is prepared using buffer solution in place of the enzyme extract **[6].**

- **Peroxidase** **(POX) Activity Determination:**

To assess peroxidase activity, a solution of 10 ml was prepared, comprising 5.8 ml of 50 mM phosphate buffer at pH 7.0, 200 µl of enzyme extract, 2 ml of 20 mM H_2_O_2_, and an additional 2 ml of 20 mM pyrogallol. The increase in absorbance resulting from pyrogallol oxidation was measured using a UV-spectrophotometer at 470 nm over 60-second duration. A blank sample was created by replacing the enzyme extract with buffer. In the enzyme assay, the initial volume (at zero time) served as the blank. The enzyme activity was quantified using the formula (∆ × T v × 60 min) / (t × v × F. Wt.), where ∆ represents the absorbance change during incubation, T v is the total volume of filtrate, t is the incubation time in minutes, v is the total volume of filtrate used for incubation, and F. Wt. denotes the fresh weight of the sample **[7].**

- **Polyphenol Oxidase (PPO) Activity Determination:**

The Polyphenol oxidase enzyme activity was determined following the method outlined by **[8].** The enzyme-substrate mixture consisted of 1.0 ml of enzyme extract, 1.0 ml of 0.2 M sodium phosphate buffer at pH 7.0, 10 ml of 0.001 M catechol, and 3.0 ml of distilled water. Absorbance was measured at 495 nm every 60 seconds, and the change in optical density was recorded. To establish enzyme activity, a blank was created by substituting the isolated enzyme with a buffer solution. Enzyme activity was quantified using the formula (∆ × T v × 60 min) / (t × v × F. Wt.), where ∆ represents the difference in absorbance before and after incubation, T v is the total volume of the filtrate, t is the incubation time in minutes, v of the filtrate for incubation is the total volume, and F. Wt. denotes the fresh weight of the sample. The results were subjected to statistical analysis as per the standard procedure **[9].**

1. **Extraction and Quantification of Soluble Carbohydrates:**

- **Extraction Procedure:**

Following the drying of plant tissue at 60°C until a constant dry weight was attained, the tissue was finely powdered. One gram of the powder slated for analysis was placed in a 100 ml capacity conical flask. To this, 5 ml of 2% phenol water and 10 ml of 30% trichloroacetic acid were added. After thorough shaking, the mixture was left overnight before filtration. The resulting filtrate was adjusted to a final volume of 50 ml **[10].**

- **Determination of Soluble Carbohydrates:**

For the quantification of soluble carbohydrates, the anthrone technique, as outlined by **[11]**, was employed. In this method, 10 ml of the extract was combined with 2 g of activated charcoal and vigorously shaken for 15 minutes. The mixture was then filtered through Whatman No.1 filter paper to obtain a clear filtrate. Distilled water was added in bulk to the filtrate. Subsequently, 2 ml of the dialyzed filtrate was transferred to a test tube, and 4 ml of a freshly prepared anthrone reagent (comprising 2 g of anthrone per 1 ml of pure 95% sulfuric acid) was added. The test tube was placed in a boiling water bath for 3 minutes. The resulting color was measured using a spectrophotometer at a wavelength of 620 nm. To calibrate the device to zero optical density (O.D.), a blank mixture containing distilled water and anthrone reagent was employed.

1. **Extraction and Quantification of Water-Soluble Proteins**

- **Extraction Procedure:**

To extract water-soluble proteins, the plant tissues were dried at 60°C until a consistent dry weight was achieved. The dried tissues were finely powdered, and one gram of this powder was introduced into a cone. Subsequently, 2% phenol water (5 ml) was added, mixed with 10 ml of distilled water. The reaction mixture was then filtered, and the resulting filtrate was adjusted with distilled water to reach a final volume of 50 ml.

- **Determination:**

According to **[12]** using casein as a standard protein.

- - - - - **Reagents** `

Solution (A): 2% (Na2CO3) in 0.1 N (NaOH).

Solution (B): 0.5 g (CuSO4) in 1% sodium potassium tartrate.

Solution (C): 50 ml of solution (A) were mixed with 1 ml of solution (B), mixing of the two solutions was done just before the protein determinations.

Solution (D): This solution was prepared by diluting folin reagent (BDH) with distilled water in the proportion of 1:3 (v/v).

- - - - - **Method**

In a test tube, 1 ml of plant sample extracts designated for protein analysis was mixed with 5 ml of solution (C). Following thorough mixing, the tubes were allowed to stand undisturbed for ten minutes. Subsequently, 0.5 ml of solution (D) was swiftly introduced and blended with the tube. The mixture was then left to stand for an additional 30 minutes, after which the optical density (O.D.) of the resulting color was measured at a wavelength of 750 nm.

**References**

[1] Vernon L P and Selly G R 1966 The chlorophylls. (Acad. Press, New York, London

[2] Lichtenthaler H, Buschmann C, Döll M, Fietz H-J, Bach T, Kozel U, Meier D and Rahmsdorf U 1981 Photosynthetic activity, chloroplast ultrastructure, and leaf characteristics of high-light and low-light plants and of sun and shade leaves *Photosynthesis research* **2** 115-41

[3] Singleton V L, Orthofer R and Lamuela-Raventós R M 1999 *Methods in enzymology*: Elsevier) pp 152-78

[4] Bates L, Waldren R a and Teare I 1973 Rapid determination of free proline for water-stress studies *Plant and soil* **39** 205-7

[5] Kong F, Hu W, Chao S, Sang W and Wang L 1999 Physiological responses of the lichen Xanthoparmelia mexicana to oxidative stress of SO2 *Environmental and Experimental Botany* **42** 201-9

[6] Aebi H 1984 *Methods in enzymology*: Elsevier) pp 121-6

[7] Castillo F J, Penel C and Greppin H 1984 Peroxidase release induced by ozone in Sedum album leaves: involvement of Ca2+ *Plant physiology* **74** 846-51

[8] Matta A and Dimond A 1963 Symptoms of Fusarium wilt in relation to quantity of fungus and enzyme activity in tomato stems *Phytopathology* **53** 574-&

[9] Snedecor G W and Cochran W G 1989 Statistical methods, 8thEdn *Ames: Iowa State Univ. Press Iowa* **54** 71-82

[10] Said A, Naguib M and Ramzy M 1964 Sucrose determination as a means of estimation of the draw back tax on exported Halawa Tehinia *Bull. Fac. Sci. Cairo Univ* **39** 207-16

[11] Umbreit W 1957 Manometric techniques

[12] Lowry O H, Rosebrough N J, Farr A L and Randall R J 1951 Protein measurement with the Folin phenol reagent *Journal of biological chemistry* **193** 265-75
